# Combination of advanced nano-Fenton process and sonication for destruction of diclofenac and variables optimization using response surface method

**DOI:** 10.1038/s41598-022-25349-1

**Published:** 2022-12-05

**Authors:** Kamyar Yaghmaeian, Nader Yousefi, Amin Bagheri, Amir Hossein Mahvi, Ramin Nabizadeh, Mohammad Hadi Dehghani, Rana Fekri, Behrouz Akbari-adergani

**Affiliations:** 1 Center for Water Quality Research, Institute for Environmental Research, Tehran University of Medical Sciences, Tehran, Iran; 2 Department of Environmental Health Engineering, School of Public Health, Tehran University of Medical Sciences, Tehran, Iran; 3 Department of Health, Safety, and Environment, School of Public Health and Safety, Shahid Beheshti University of Medical Sciences, Tehran, Iran; 4 Center for Solid Waste Research, Institute for Environmental Research (IER), Tehran University of Medical Sciences, Tehran, Iran; 5 Department of Environmental Health Engineering, School of Public Health and Safety, Shahid Beheshti University of Medical Sciences, Tehran, Iran; 6 Nanotechnology Products Laboratory, Food and Drug Laboratory Research Center, Food and Drug Organization, Ministry of Health and Medical Education, Tehran, Iran

**Keywords:** Environmental chemistry, Environmental impact

## Abstract

Diclofenac (DCF) as a non-steroidal pharmaceutical has been detected in aquatic samples more than other compounds due to its high consumption and limited biodegradability. In this study, ultrasound waves were applied along with an advanced nano-Fenton process (US/ANF) to remove DCF, and subsequently, the synergistic effect was determined. Before that, the efficiency of the US and ANF processes was separately studied. The central composite design was used as one of the most applicable responses surface method techniques to determine the main and interactive effect of the factors influencing DCF removal efficiency in US/ANF. The mean DCF removal efficiency under different operational conditions and at the time of 1–10 min was obtained to be about 4%, 83%, and 95% for the US, ANF, and US/ANF, respectively. Quadratic regression equations for two frequencies of US were developed using multiple regression analysis involving main, quadratic, and interaction effects. The optimum condition for DCF removal was obtained at time of 8.17 min, H/F of 10.5 and DCF concentration of 10.12 at 130 kHz US frequency. The synergy index values showed a slight synergistic effect for US/ANF (1.1). Although the synergistic effect of US/ANF is not very remarkable, it can be considered as a quick and efficient process for the removal of DCF from wastewater with a significant amount of mineralization.

## Introduction

Owning to the wide consumption of pharmaceuticals, although in very trace concentrations, they have been detected in various aquatic samples including water bodies, municipal wastewater, and hospital effluents^1–3^. Therefore, their presence has raised concerns about adverse environmental and health effects^4,5^. Diclofenac (DCF) with a chemical formula of C_14_H_11_Cl_2_NO_2_ is a nonsteroidal, analgesic, and anti-inflammatory drug. Due to the high consumption of DCF (around 1000 tons/year) and limited biodegradability in the wastewater treatment plant, it has been detected in aquatic samples more than in other compounds^6,7^. Based on the most recent data (2016–2017) provided by the Food and Drug Organization of the Iranian Health Ministry about 55 tons of DCF were produced, imported, and distributed in the country^8–10^. Conventional wastewater treatment plants are not able to remove DCF and other pharmaceuticals completely^11–13^. A huge reduction in the number of vultures in Pakistan and India due to kidney failure following feeding on contaminated animals with DCF represents the risk of its presence in the environment^14–16^.

Most of the recent research has focused on the application of non-biological methods like advanced oxidation processes (AOPs) for pharmaceuticals and other pollutants removal from aqueous solutions^17–22^. Unlike other wastewater treatment methods such as adsorption^23^, chemical coagulation^24^, and membrane processes^25^, AOPs are able to eliminate organic pollutants without changing their phase^12^. Among them, the Fenton process has increasingly been considered owning to the simple operation and fast reaction, low toxicity of the used catalyst, feasibility to use as a complementary treatment, and economic considerations^26–28^. Generally, the conventional Fenton process needs large amounts of catalyst (i.e. Fe^2+^) to produce sufficient OH radicals. However, it has been proved that the efficiency of the heterogeneous Fenton process using zero-valent iron as a catalyst is remarkable compared to conventional Fenton. Since the process has fast reactions and high efficiency for contaminants removal, some authors named it advanced Fenton (AF)^29–31^. After entering ZVI into the water, its surface becomes oxidized, thereby generating ferrous iron, which will induce Fenton's reaction indirectly. Besides, when sufficient dissolved oxygen is available, hydrogen peroxide, which may reduce the consumption of oxidants, is also produced^32^. Another method that is used for the removal of some organic contaminants and has been known as a green treatment with no chemical addition is sonochemical processes or the use of ultrasonic (US) waves^33^. Several applications of ultrasound have been reported^34–41^. Because of the cavitation and vibration of the formed bubbles, this method can promote mechanical, thermal, or chemical effects in an aqueous environment. Furthermore, owing to the dramatic oscillations and highly variable gas pressure inside the bubbles, a phenomenon similar to ionization is prompted in the gas volume, causing the formation of free radicals and increasing their concentration in the surrounding aqueous environment, thereby affecting the treatment of contaminants^42–44^.

Ultrasound would be hybrided with the heterogeneous ANF system, achieving a significant removal of organic compound. But based on the our best knowledge, it seems that the previous studies are unsuificient yet. Therefore, in the present work, a US/ANF system was suggested and surveyed for the removal of a highly used diclofenac drug. In our previous work, the efficiency of nanoparticle zero-valent iron as catalyst was examined. The process was named the advanced nano-Fenton process (ANF)^6^. In this study, initially, the efficiency of the US processes alone in removing DCF from aqueous solutions was studied. Since ANF and US produce active oxidative species such as OH°/OOH°, the main aim of our work was to apply US waves along with advanced nano-Fenton process (US/ANF) to remove DCF, so that the synergistic effect or other paths and possible reactions would be determined in response to the combined usage of these AOPs. By considering operating variables (initial concentration, pH, molar ratio of hydrogen peroxide to iron, and reaction time) and testing their different values, the optimal conditions for the incidence of the synergistic effect between these two processes were determined by specifying the DCF removal efficiency.

## Materials and methods

### Materials

Nanoparticles of zerovalent iron (35–45 nm, 99.5%) and sodium diclofenac were provided from Sigma-Aldrich. Tert-butanol (99.5%) as an agent of quenching and scavenging of radicals of the reactions (generated in oxidation process (hydroxide radicals)), methanol (high purity, 96%), hydrogen peroxide (30% w/v), MQuant strips for measuring the residual amounts of hydrogen peroxide, sodium chloride, and sodium carbonate (analytical purity) for IC mobile phase and preparation of stock and standard solutions, acetic acid as HPLC mobile phase and ultra-filtrated water, phosphoric acid and sodium hydroxide for pH adjustment were purchased from Merck Millipore. Besides, distilled water (Electrical Conductivity ~ 1.2 ± 0.2 S/m) produced by an Ultrapure filtration device (Human) was used.

### Experiments procedures

**Figure 1:**
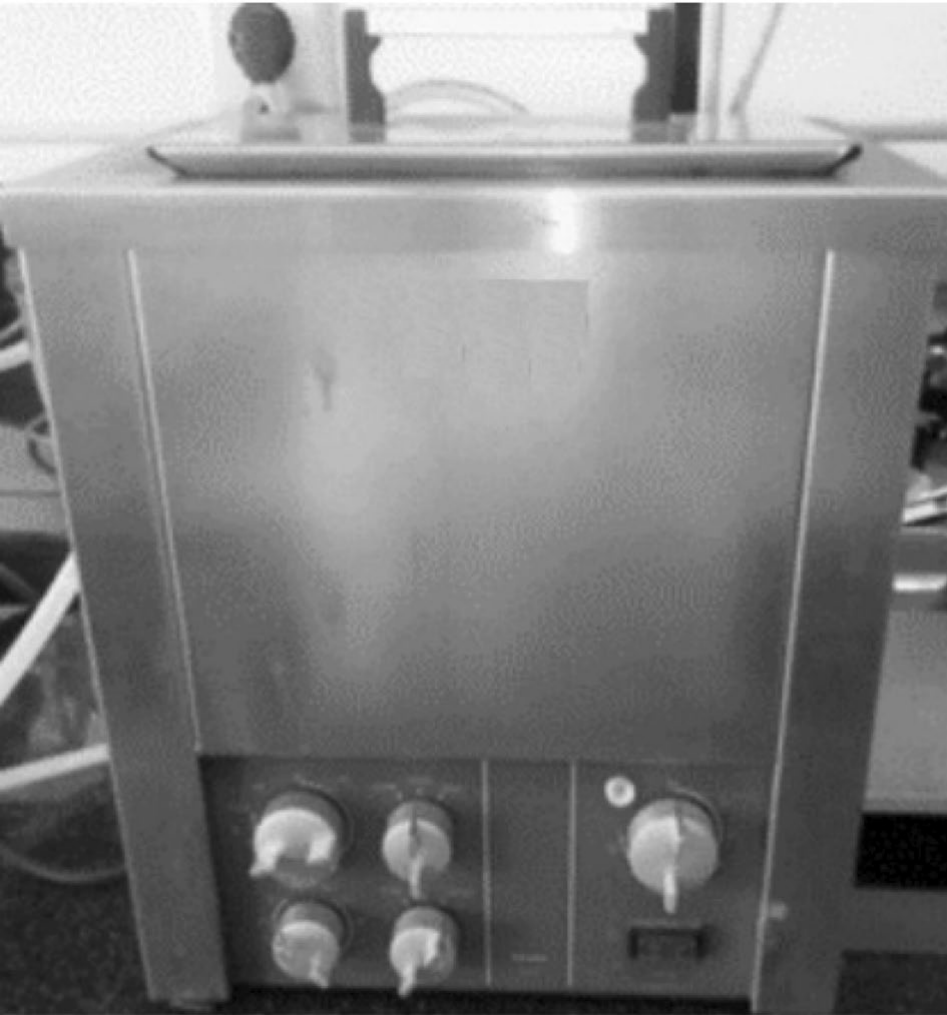
Photograph of bath sonicator used for the removal of DCF.

The method used in this study has been taken from previous study^6^. The summarized method has been mentioned in the following. The reactions were started with the addition of hydrogen peroxide into the prepared samples or turning on the US device after increasing certain amounts of nanoparticles of ZVI (NZVI) and DCF to distilled water. All experiments have been carried out in an Erlenmeyer flask with a volume of 100-mL. Mild aeration was done using a diffuser of microporous air for mixing the contents of the reactor because of more feasible in large-scale wastewater treatment plants in comparison with magnetic stirrers and considering the role of soluble oxygen in the preservation of hydroxyl radicals produced in Fenton and sonolysis processes^43^. During the US process, the temperature was controlled at 25 ± 2 °C. 100 µL of each sample was withdrawn at certain intervals and adding 100 µL of tert-butanol 2 mol/L (for quenching the reaction) and then, the samples were injected into HPLC for measuring DCF residues. Afterward, the factors influencing the DCF removal efficiency were investigated. The ultrasound instrument (Fig. 1) used in this research was a bath sonicator made of stainless steel, which had two transducers. The level of acoustic energy generated by the device was measured using the calorimetric method^45^. The device specifications are provided in Table 1.

**Table 1:** Specifications of bath sonicator.

Apparatus type	Elma TI-H-5 (Germany)
Electrical power	500 W
Frequency	35 and 130 kHz
Transducer no.	2 (with a 5 cm diameter)
Tank volume	3.5 L
Weight	10.5 kg

As the sonicator used in this study was equipped with two frequencies of 35 and 130 kHz, the effect of other influential variables including pH, irradiation time, and initial concentration of DCF was investigated at both frequencies.

### Experimental design

#### US processes alone

In order to determine the major effects of different parameters that influence the drug removal efficiency in US processes, a full factorial design with 3 replicates was applied using R software (3.1.0). In the study of the US process, the effective variables of pH, irradiation time (iTime), the initial concentration of the DCF (Conc), and the frequency of waves (Freq) were examined. Table 2 indicates the High and low levels of the variables.

**Table 2:** Levels and factors of factorial designs for the US process.

Process	Factors	Low level	High level
a. Ultrasound process (US)	iTime (min)	1	10
	Conc (mg L^−1^)	5	15
	pH	3	11
	Freq (kHz)	35	130

#### US/ANF processes

Central composite design (CCD) was applied as one of the most frequently used techniques among RSM designs to determine the main and interactive effects of the factors influencing DCF removal efficiency in the hybrid US/ANF process^35^. The initial concentration of DCF (Conc), the reaction time (iTime), and the molar ratio of hydrogen peroxide to NZVI (H/F) were studied within the ranges of 5–15 mg/L, 1–10 min, and 5–15, respectively. As the sonicator utilized in this study was only able to generate two separate frequencies, thus CCD was performed in two individual phases (35 and 130 kHz as indicators for low and high frequencies ultrasound waves respectively). All of the variables were coded across five levels and the experiments were carried out based on the random arrangement. Table 3 presents the real values corresponding to each code.

**Table 3:** Factors and coded levels of independent variables for US/ANF process at 35 and 130 kHz.

Factor	Variable	Variable level				
		− 2	− 1	0	+ 1	+ 2
Conc (mg L^−1^)	X_1_	5	7.5	10	12.5	15
Time (min)	X_2_	1	3.25	5.5	7.75	10
(H/F)	X_3_	5	7.5	10	12.5	15

### Analytical methods

DCF residual concentration was determined by high-pressure liquid chromatography (HPLC) device, KNAUER Model, with a C18 column, the particle diameter of 5 µm, and dimensions of 250 × 4.6 mm at the wavelength of 278 nm (JRC91354 EUR 26902 EN standard). A mixture of methanol and water solution with a 60:40 ratio (containing 0.1% acetic acid) with a flow rate of 1 mL/min along the analysis was used as the mobile phase. The drug measurement was conducted under working conditions at 30 °C and column pressure of about 18 MPa. For diclofenac measurement, Microliter Syringes, Hamilton (Reno, CA, USA), Dionex Autotrace AT280 automated SPE system (Thermo Scientific, Waltham, MA, USA), TurboVap II (Caliper Life Science, Mountain View, CA, USA) Vortex Genius, Ika, Staufen, Germany was used. To measure the level of the chlorine liberated in the sample in response to DCF degradation in the US/ANF process, the Metrohm chromatography device, 850 professional IC was used. The utilized column Metrosep a Supp 1 with a particle diameter of 9 µm and dimensions of 250 × 4.6 mm, the detergent solvent of sodium carbonate 0.35 M, the flow rate of 1 mL/min, the temperature of 29 °C, and pressure of 8.9 MPa were the specifications of the experiments.

The limits of detection and quantification were estimated by analysing 15 different blank samples. The mean value of blank samples (b) and the RSD served for LOD and LOQ estimation, in accordance with the following equations:

$${\text{LOD }} = {\text{ b }} + {\text{ 3SD}}$$

$${\text{LOQ }} = {\text{ b }} + { 1}0{\text{SD}}$$

The degree of mineralization was determined through TOC analysis and with the TOC-CSH device, Schimatdzu. The extent of hydrogen peroxide consumption across different reactions was determined using calorimetry strips (MQuant) and through dilution. The colorimetric method of 1,10-phenanthroline was used to determine Ferrous iron in solutions^46^.

## Results and discussion

### US process

**Figure 2:**
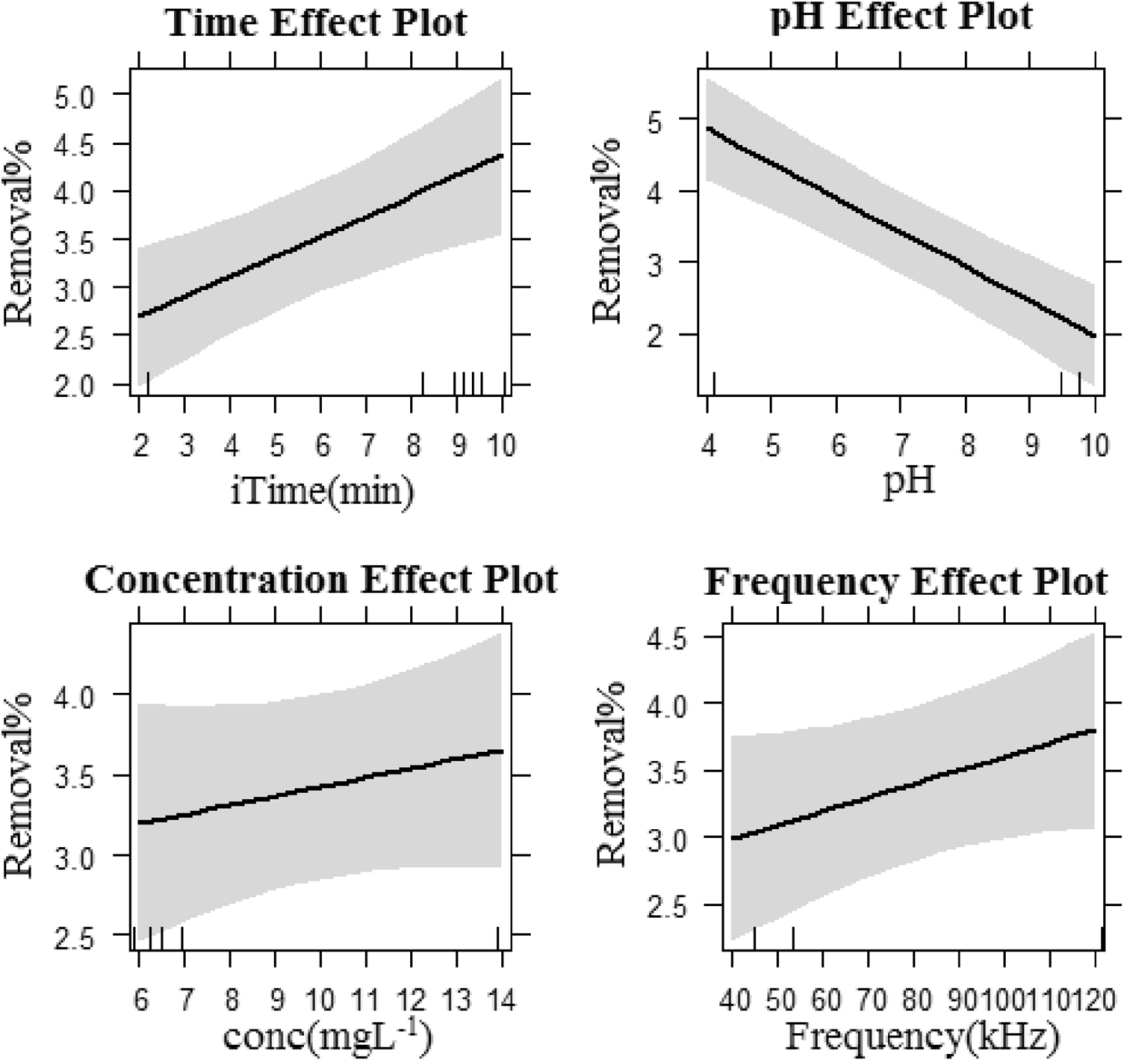
Main effect of individual factors for DCF removal efficiency in US process.

**Figure 3:**
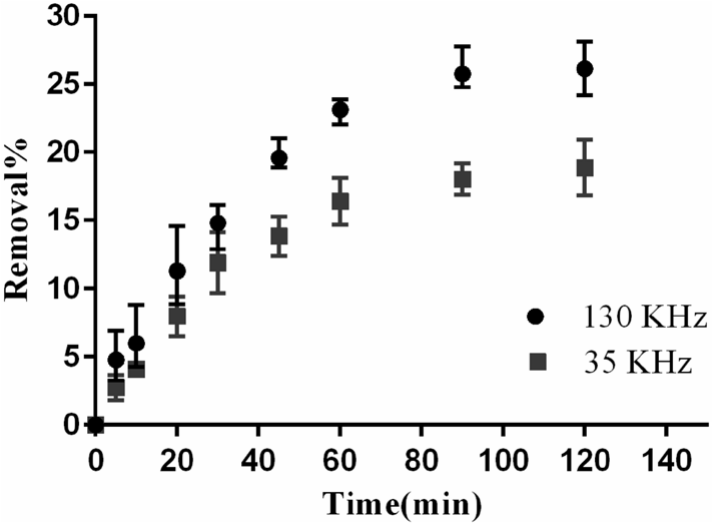
Removal efficiency of DCF at an extended period of US irradiation and two frequencies (DCF concentration = 5 mg/L and pH = 3).

In the US process alone, the mean DCF removal efficiency under different operational conditions and within 1–10 min was obtained to be below 4% (low value in comparison to other AOPs). The electric power consumed by the ultrasound device was 500 W, while the mean acoustic power exerted to the solution at the frequencies of 35 and 130 kHz was measured to be 24 and 37 W/L, respectively. In indirect ultrasound systems such as bath sonicator, the extent of changes in the input electric power is not in line with the acoustic power generated in the solution, where the acoustic power is generally a constant value^47^. The low efficiency of the instrument in converting the input energy to acoustic power results in diminished production of effective radicals both at the bulk and molecular level (micro-level) of the solution. In this study, by increasing the independent variables of time, frequency, and initial concentration of DCF, the removal efficiency increased, while with the increase in the pH, the efficiency decreased (Fig. 2). Overall, at pH values lower than pKa, an organic compound gets more subject to reaction with active radicals such as hydroxyl due to being converted to a molecular state (more hydrophobic). Further, since the hydroxyl production reactions mainly occur at the molecular level and in the vicinity of cavitation bubbles, DCF removal efficiency is greater at acidic pH (below pKa = 4.15), when compared with neutral and alkaline pH^14^. As the determinant parameter of the sonolysis mechanism (mechanical oxidation and production of free radicals), the frequency can affect the removal efficiency of the target analyte, such that with the increase in frequency, the size of the bubbles shrinks, and more free radicals are produced^34^. Although in US process with frequency increase from 35 to 130 kHz the removal efficiency increased (Fig. 2), the results of full factorial design indicate that unlike the parameters of time and pH (significance level of 0.05), frequency is effective at the significance level of 0.1, which can be associated with the superior effect of mechanical degradation of DCF at the frequency of 35 kHz in comparison with oxidation in response to free radical production (Table 4). Similarly, the concentration is not significant. The same reason can be applied for the interaction between concentration and frequency. At low concentrations, free radicals (which are produced more at high frequencies sonication) react more effectively with DCF molecules; conversely, at high concentrations, the mechanical effect of cavitation bubbles (which is the dominant mechanism at low-frequency sonication) causes DCF destruction. Additionally, during times beyond the studied period, greater removal efficiency is achieved at the frequency of 130 kHz owing to the production of more free radicals and the possibility of collision between the DCF molecules and the radicals. It obviously shows that the removal of DCF improves with an enhance of ultrasonic power. The ability of ultrasonic irradiation degassing would be increased at high ultrasonic power. Then, the oxygen gas solubility would be critically declined with enhancing ultrasonic power. Therefore, the total efficacy of hydrogen peroxide was improved with an increase in ultrasonic power. In addition, the amounts of free radicals generated in the solution were enhanced at high values of ultrasonic power. Performance investigation of sonolysis in removing DCF was conducted at the two individual frequencies and under optimal conditions (acidic pH and DCF concentration of 5 mg/L) and within a longer time interval than the studied period (Fig. 3). Eventually, following 120 min, the greatest DCF removal efficiency was obtained to be about 28% at the frequency of 130 kHz.

**Table 4:** Estimated coefficients and other results of the fitted models for DCF removal using US process (full factorial design).

Process	Model terms	Estimated coefficient	*t*-value	*p*-value	Other model results
Sonolysis (US)	Intercept	3.43562	13.0414	4.92 × 10^–08^	Multiple R^2^: 0.8684; Adjusted R^2^: 0.8153; F-statistic: 17.5 on 4 and 11 DF; *p*-value: 9.65 × 10^–05^; Std. Error: 0.263
	X_1_ = time	0.93437	3.5468	0.004578	
	X_2_ = pH	− 1.91812	− 7.2811	1.58 × 10^–05^	
	X_3_ = Conc	0.30938	1.1744	0.265046	
	X_4_ = Freq	0.47437	1.8007	0.099201

### ANF process

**Figure 4:**
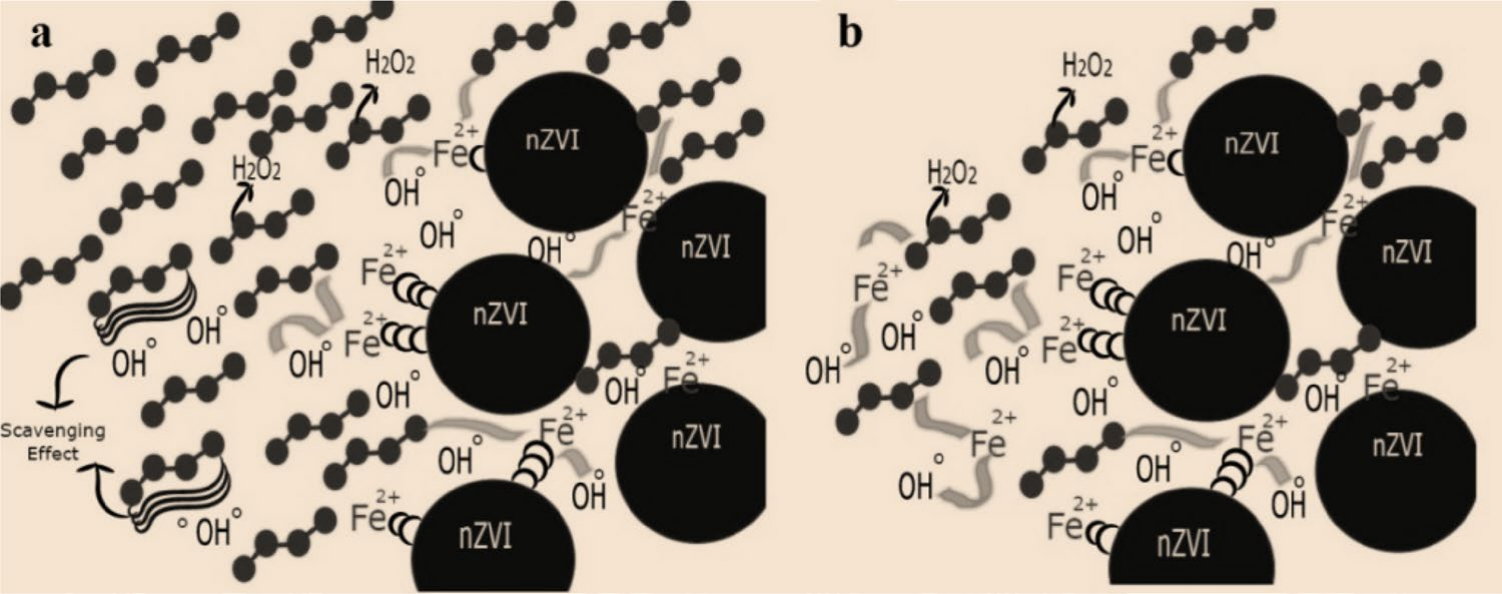
Schematic of ANF process (**a** radical scavenging effect due to the high amount of HP, **b** optimal condition without scavenging effect).

The results of our previous work showed that the average efficiency of ANF for removing DCF was over 83%. The mutual effect of hydrogen peroxide concentration on DCF removal was also discussed^6^. In Fig. 4a, an excessive amount of hydrogen peroxide and the possibility of development of radical scavenging effect along with optimal conditions and with minimum probability of radical scavenging (Fig. 4b) have been shown schematically. The high amount of hydrogen peroxide causes the conversion of radicals to molecular species in the reaction and subsequent reduction in DCF removal efficiency.

### Hybrid US/ANF

Since the main objective of the study was to investigate the efficiency of processes within a short time (1–10 min), the results indicated that the hybrid process has very high efficiency, such that the mean DCF removal efficiency at the frequencies of 35 and 130 kHz was obtained to be 94 and 96%, respectively. Under optimal conditions of both phases of the study (35 and 130 kHz), the removal efficiency of over 99% was also achieved.

#### Model fitting and statistical analysis

ANOVA was used for investigating the proposed models. Based on the results obtained at the frequency of 35 kHz (Table 5), it was found that the model had an R^2^ and adjusted R^2^ (adR^2^) of 0.95 and 0.93 respectively, suggesting the validity of the model in terms of statistics. Furthermore, the lack of fit (LOF) value was obtained to be 0.49 for a frequency of 35 kHz (Table 6). The difference between R^2^ and adR^2^ and LOF showed that the removal performance of DCF as the response variable is well accounted for by the proposed model. The results corresponding to the frequency 130 kHz showed that the R^2^ and adR^2^ values were 0.99 and 0.98, respectively with a LOF value of 0.47. Table 6 summarizes the results of the analysis of variance for both phases of the statistical study (35 and 135 kHz). In contrast to 35 kHz, the variable of DCF concentration in the suggested model for US/ANF at the frequency of 130 kHz has an acceptable significance level (0.002). Quadratic regression equations for both phases (two frequencies) were developed using multiple regression analysis on coded data involving main, quadratic, and interaction effects as follows:

1 $${\text{Y}}_{{({35})}} = {97}.0{3} + {15}.{\text{11X}}_{{1}} + {3}.{\text{53X}}_{{2}} - {12}.{\text{28X}}_{{1}}{^{{2}}} - {5}.{\text{48 X}}_{{3}}{^{{2}}} - { 5}.{\text{69X}}_{{1}} {\text{X}}_{{2}}$$

2 $${\text{Y}}_{{({13}0)}} = {98} + {12}.{\text{89X}}_{{1}} + {4}.{\text{12X}}_{{2}} - {1}.{\text{11X}}_{{3}} - {13}.{\text{41X}}_{{1}}{^{{2}}} - {3}.{\text{35X}}_{{2}}{^{{2}}} - {1}.{\text{37X}}_{{3}}{^{{2}}} - {6}.0{\text{4X}}_{{1}} {\text{X}}_{{2}}$$

**Table 5:** Estimated coefficients of the fitted polynomial model for DCF removal using ANF at 35 and 130 kHz.

Model terms	Coefficient Estimate		Std. Error		*t*-value		*p*-value	
	35 kHZ	130 kHZ	35 kHZ	130 kHZ	35 kHZ	130 kHZ	35 kHZ	130 kHZ
Intercept	97.039	98.002	0.50798	0.18367	191.0306	533.5793	< 2.2 × 10^–16^	< 2.2 × 10^–16^
X_1_ = Time	15.113	12.896	0.93072	0.33652	16.2386	38.3226	1.35 × 10^–12^	< 2.2 × 10^–16^
X_2_ = H/F	3.531	4.126	0.89067	0.32204	3.9655	12.8127	0.000829	8.50 × 10^–11^
X_3_ = Conc	− 0.756	− 1.112	0.89067	0.32204	− 0.8492	− 3.4551	0.406348	0.002652
X_1_X_2_	**− 5.692**	**− 6.048**	2.08692	0.75457	− 2.7277	− 8.0162	0.013365	1.63 × 10^–07^
X_1_X_3_	1.417	− 0.236	2.08692	0.75457	0.6792	− 0.3131	0.505182	0.757622
X_2_X_3_	1.833	0.190	1.93233	0.69868	0.9488	0.2733	0.354651	0.787545
X_1_^2^	− 12.283	− 13.412	1.37155	0.49591	− 8.9562	− 27.0459	3.01 × 10^–8^	< 2.2 × 10^–16^
X_2_^2^	− 0.996	− 3.359	1.33039	0.48103	− 0.7487	− 6.9847	0.463188	1.18 × 10^–06^
X_3_^2^	− 5.487	− 1.374	1.33039	0.48103	− 4.1248	− 2.8582	0.000576	0.01006

Significant values are given in bold.

**Table 6:** Analysis of variance (ANOVA) for the fitted polynomial models (35 and 130 kHz) for DCF removal using US/ANF.

Response: DCF removal (%)		Df	Sum Sq	Mean Sq	*F* value	Pr(> F)
35 kHz	First-order	3	1084.5	361.5	93.3796	1.49 × 10^–11^
	Two-way interaction	3	34.07	11.36	2.934	0.05984
	Pure quadratic	3	405.93	135.31	34.9519	6.18 × 10^–08^
	Residuals	19	73.55	3.87		
	Lack of fit	5	18.21	3.64	0.9214	0.49565
	Pure error	14	55.34	3.95		
130 kHz	First-order	3	832.42	277.472	548.2421	2.20 × 10^–16^
	Two-way interaction	3	32.61	10.87	21.4771	2.53 × 10^–06^
	Pure quadratic	3	421.56	140.519	277.6433	7.29 × 10^–16^
	Residuals	19	9.62	0.506		
	Lack of fit	5	2.47	0.493	0.9657	0.4712
	Pure error	14	7.15	0.511		

### Evaluation of variables effects

**Figure 5:**
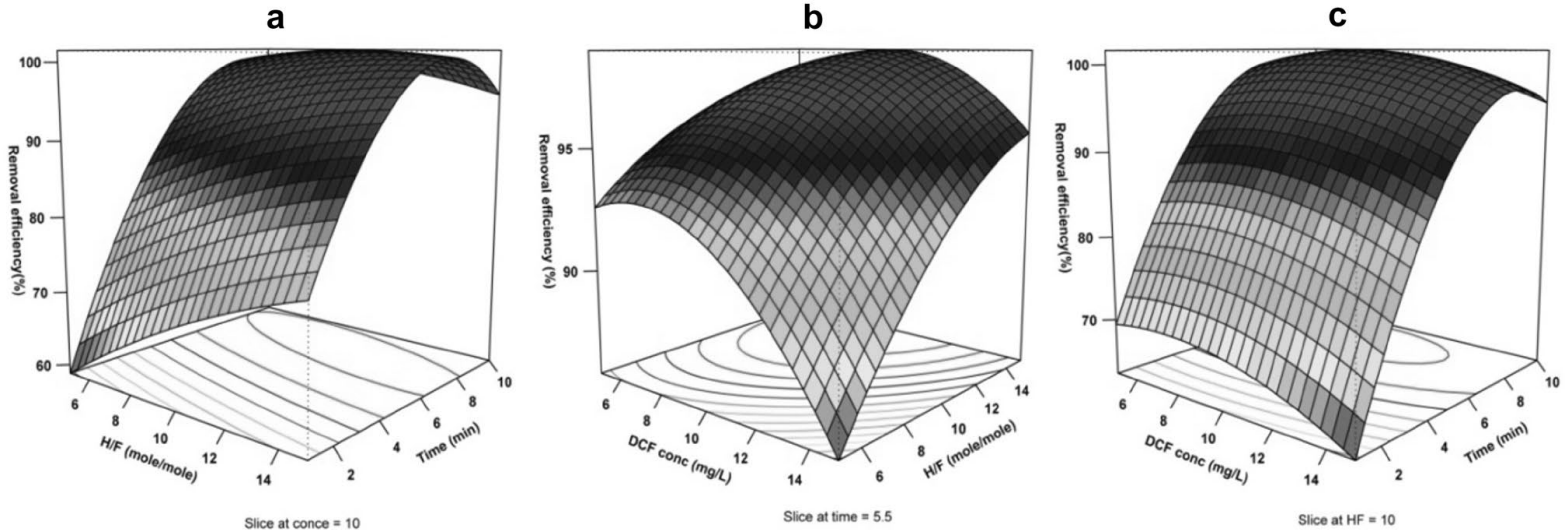
3D response surface plots for DCF removal at 35 kHz. The effect of (**a**) H/F and time, (**b**) initial DCF concentration and H/F, (**c**) initial DCF concentration and time.

**Figure 6:**
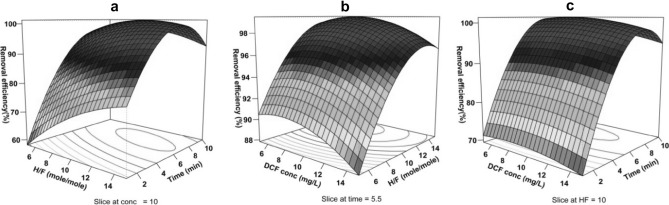
3D response surface plots for DCF removal at 130 kHz. The effect of (**a**) H/F and time, (**b**) initial DCF concentration and H/F, (**c**) initial DCF concentration and time.

**Figure 7:**
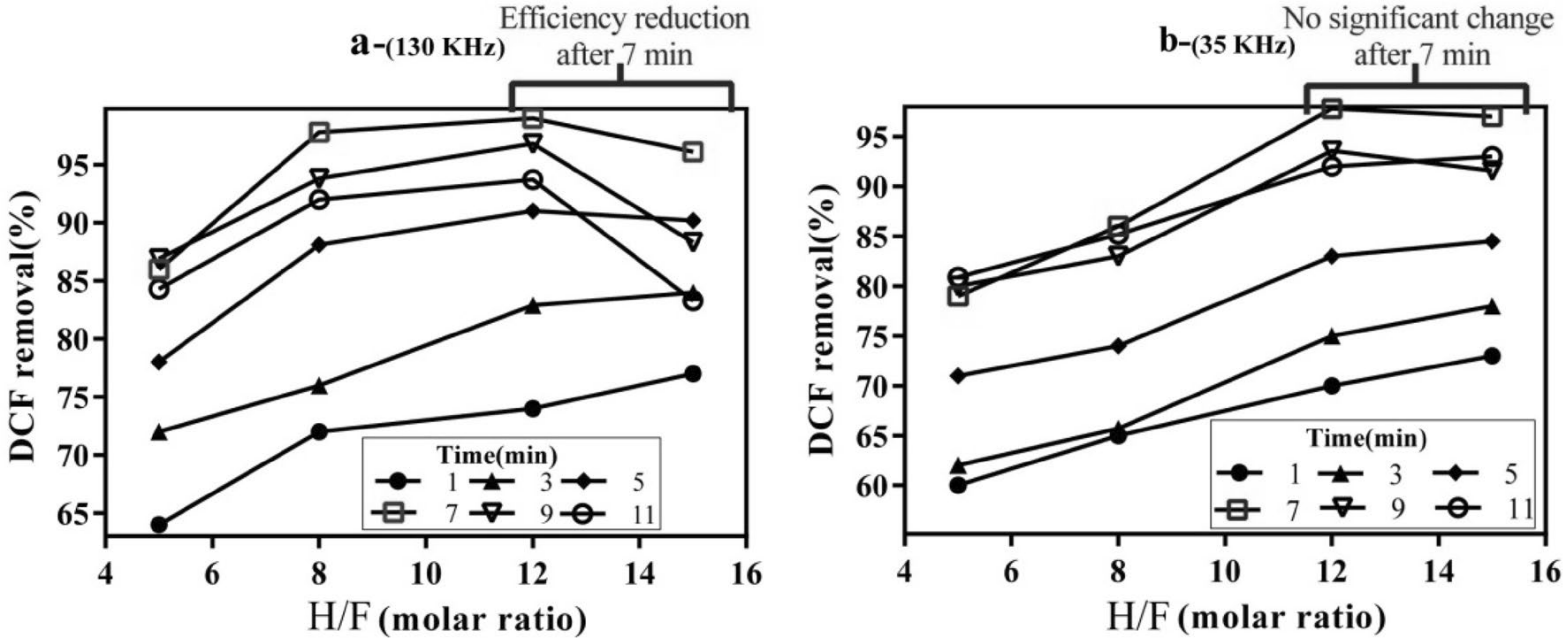
Efficiency variations of DCF removal in different H/F ratios and reaction times (**a** 130 kHz and **b** 35 kHz).

Graphic representation of the presented models (Eqs. 1 and 2) are shown in Figs. 5a–c and 6a–c in the form of a surface plot. The concurrent effect of the independent variables on the response (DCF removal efficiency) was examined. Expectedly, in both models with an increase in the reaction time, DCF removal efficiency also increases, though the effect of the initial concentration of the drug was different so that at the lowest and high concentrations it has an inverse effect on the DCF removal efficiency. Based on the outputs of models and applying an excel solver for estimations, the optimal amount of each parameter was achieved at both frequencies (Table 7). The model results obtained from regression analysis showed that the significance level of the interaction between the two variables of time and H/F is greater than that of the interaction among other variables (Bold number in Table 5). According to Fig. 7a,b, at both frequencies, within the early minutes of the reaction (t ≤ 5 min, solid symbols), with the increase in H/F, greater efficiency of DCF removal in comparison with the middle and later times of the reaction (t ≥ 7 min, empty symbols) was achieved. Considering the low concentrations of DCF, at the early minutes of the reaction with the increase in the level of hydrogen peroxide, Fenton process efficiency increases and causes DCF removal up to an acceptable level. However, during middle times of the reaction (about 7 min), the effect of H/F increase on removal efficiency gradually declines, where the increase in this ratio up to middle values (the optimal point) results in increasing the efficiency of DCF removal, while at higher H/F, it does not show any significant effect on the response variable. This might be due to the presence of an excessive amount of hydrogen peroxide and the development of radical scavenging conditions (Fig. 4a). At a frequency of 130 kHz, DCF removal efficiency even declines to some extent following the optimal point of molar ratio (H/F ≈10) (Fig. 7b). As production of radicals in response to cavitation (chemical effects) at the frequency of 130 is predominant about mechanical and micro-mixing effects (frequency of 35), the excessive amount of hydrogen peroxide in the experimental reactor causes removal of hydroxyl radicals, and thus the drug removal efficiency decreases, though these differences are very minor^42^. At the frequency of 35, although the negative scavenging effect of the extra hydrogen peroxide in the ANF process has caused the efficiency not to increase that much, due to the physical effect of the cavitation of the bubbles through developing microscopic mixing and the possibility of development of contact between the radicals and the target contaminant, the radical scavenging effect has been inhibited to some extent^37,42^. Therefore, the removal efficiency at higher H/F ratios has not diminished, unlike the frequency of 130 (Fig. 7b).

**Table 7:** Optimal value for independent variables of US/ANF model at 35 and 130 kHz.

Frequency	Independent variables		
	Time (min)	H/F	DCF conc (mg/L)
35 kHz	8.17	10.50	10.12
130 kHz	7.46	11.33	7.89

### Synergistic effect

To identify the synergistic DCF degradation achieved in the US/ANF process, comparative experiments in the separated and combined processes were conducted at optimized values of variables and a fixed value of DCF concentration. The mean differences between DCF removal in the ANF and US/ANF process at optimized conditions reached 9.2 and 10.7% at 35 and 130 kHz, respectively (data not shown). In the case of applying multiple processes, synergy index is a common quantitative method to evaluate any synergistic effect which is as follow:

3 $$\mathrm{Synergy \; index}=\frac{Removal\%(US/ANF)}{Removal\%\left(US\right)+Removal\%(ANF)}$$

The index values > 1 indicate the hybrid process exceeds the sum of the separate processes^48^. Substituting mean values of removal efficiency for each process in Eq. (3) yields synergy indexes of 1.08 and 1.11 at 35 and 130 kHz, respectively. The index values showed a slight synergistic effect for the hybrid process (US/ANF).

The destruction of DCF takes place on the NZVI surface (heterogeneous Fenton) or maybe the outcome of the homogeneous Fenton process in the bulk solution (the reaction of hydrogen peroxide and the dissolved ferrous ion)^28^. To determine the contribution of homogeneous oxidation to the DCF removal, the amount of Fe^2+^ in the experiment flask of the optimized US/ANF process was measured. Afterward, the homogenous Fenton process using the optimum amount of hydrogen peroxide and the measured amount of ferrous ion (0.21 mg/L) was simulated. The removal efficiency of DCF after 10 min was less than 4% which is not comparable with the removal efficiency of the US/ANF process (≈ 99%). Other studies on hybrid and heterogeneous processes have shown similar results from the higher contribution of heterogeneous Fenton^28,46^.

The reaction series of heterogeneous Fenton-like enhanced by US was proposed where the symbol ≡ shows the iron species bound to the NZVI surface and the symbol))) indicates US irradiation^28^:

$$\equiv {\text{Fe}}^{{{3} + }} + {\text{ H}}_{{2}} {\text{O}}_{{2}} \to \, \equiv {\text{Fe}}\left( {{\text{OOH}}} \right)^{{{2} + }} + {\text{ H}}^{ + }$$

$$))){\text{Fe}}\left( {{\text{OOH}}} \right)^{{{2} + }} + \, ))) \, \to \, \equiv {\text{Fe}}^{{{2} + }} + {\text{ HO}}_{{2}}{^{\circ}}$$

$$\equiv {\text{Fe}}^{{{2} + }} + {\text{ H}}_{{2}} {\text{O}}_{{2}} \to {\text{ Fe}}^{{{3} + }} + {\text{ OH}}^{ - } + {\text{ OH}}^{\circ}$$

One reason why the synergistic effect occurred in the hybrid process can be explained by the increase of second reaction kinetics due to the faster dissociation of Fe(OOH)^2+^ complex via sonication. The next reason is that not only irradiation of US causes faster decomposition of hydrogen peroxide compared to the absence of US^39^ but also, US irradiation of a blank aqueous solution (water molecules) might enhance the production of hydrogen peroxide and active radical species by itself based on the following reactions^32^:

$${\text{H}}_{{2}} {\text{O}} + ))) \, \to {\text{OH}},^{\circ} + {\text{H}}^{\circ}$$

$${\text{OH}} \to {\text{H}} + {\text{H}}_{{2}} {\text{O}}^{\circ}$$

$${\text{2 OH}}^{\circ} \to {\text{H}}_{{2}} {\text{O}} + {\text{O}}^{\circ}$$

$${\text{2 OH}}^{\circ} \to {\text{H}}_{{2}} {\text{O}}_{{2}}$$

$${\text{2 H}}^{\circ} \to {\text{H}}_{{2}}$$

Another explanation might be the occurrence of the micro-mixing phenomenon due to the US bubbles cavitation which provided the possibility of collisions between radicals and DCF molecules and also hydrogen peroxide and formed iron ions at the metal–liquid interfaces^28,37^.

The reactions of heterogeneous corrosion in the interphase of Fe-water could be critically increased in the exposure of US irradiation. The perpendicular-shape generated to the surface Fe0 due to the water microjets as a function of collapse of sonochemical cavitation bubbles, might be lead to an the surface erosion and wide pitting. In addition, shockwave generated from US can enhance the mass transfer of the reactions of heterogeneous corrosion caused by microscopic turbulence within the thin films creating in the Fe nanoparticle surrounding.

### TOC abatement and dechlorination

**Figure 8:**
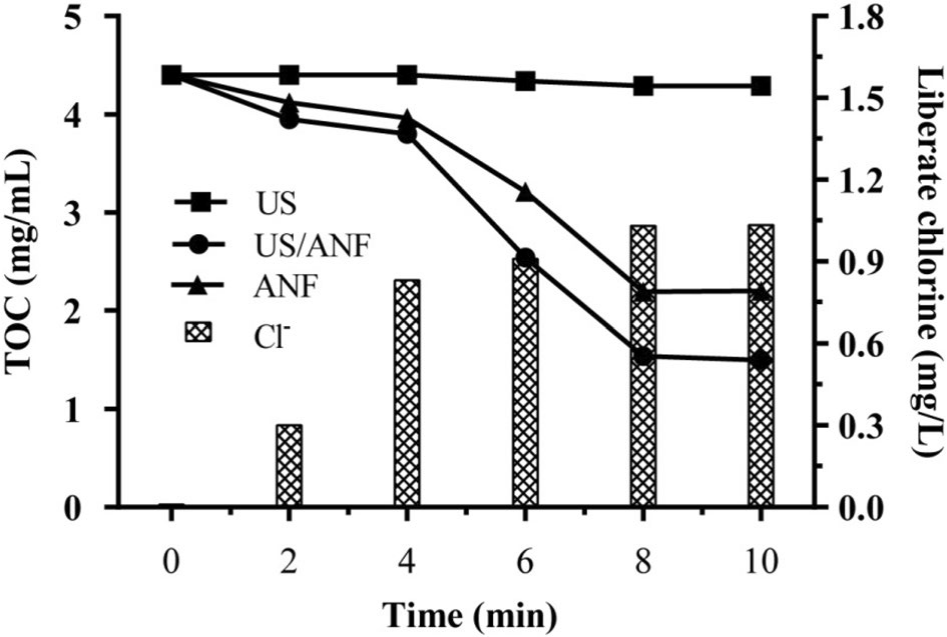
TOC reduction rate in US, ANF and US/ANF and Dechlorination rate in US/ANF (in optimum condition: initial concentration of DCF: 8 mg/L; H/F: 11, frequency 130 kHz).

The mineralization of DCF in terms of TOC removal was investigated. Figure 8 indicates the extent of TOC abatement in the US, ANF, and US/ANF. At the optimal variables, initial TOC (4.4 mg/L) decreased to 1.5 mg/L after 10 min representing that 66% of DCF was mineralized to HCl or CO_2_ in the US/ANF process, whereas at the same time ANF alone showed less than 54% TOC reduction. ANF process used to degrade 4-chloro-3-methyl phenol in a study indicated TOC removal of 63% after 60 min in optimum condition^49^. Furthermore, a Fenton-like process using pyrite as a catalyst applied to eliminate DCF could achieve 81% TOC removal after 5 min^50^. The difference between TOC abatement rate and drug removal efficiency is probably because of the presence of stable organic intermediates and the formation of recalcitrant by-products^14^. Also, the dechlorination of the DCF solution was evaluated. Results showed that the highest dechlorination of DCF was obtained under optimum conditions of the hybrid process which was equal to 29.4% (Fig. 8).

## Conclusion

Although the use of bath sonicator alone did not show suitable performance for removal of DCF, the synergistic effects with other treatment methods and also no addition of chemicals still encourage researchers to apply the US and conduct more experimentations. In the full factorial design of the US, only sonication time and pH were significant. The frequency was not significant due to the proper effect of DCF mechanical degradation at the frequency of 35 kHz in comparison with oxidation via free radicals. The same reason was applied for an explanation of the interaction of concentration and frequency variables which led to no significance of the concentration variable. In the ANF process, far greater efficiency was achieved in comparison with the US. In US/ANF, the DCF removal as the response variable is well accounted for by the proposed model. Quadratic regression equations for both phases (low and high-frequency the US) of the hybrid process were developed using multiple regression analysis involving main, quadratic, and interaction effects. Although the synergistic effect of US/ANF is not very remarkable, it can be considered as a quick and efficient process for the removal of DCF from wastewater with a significant amount of mineralization.

## Data Availability

The datasets used and/or analysed during the current study available from the corresponding author on reasonable request.
